# Evaluation of COVID-19 Surveillance Strategy in Ecuador

**DOI:** 10.1017/dmp.2020.326

**Published:** 2020-09-09

**Authors:** Ricardo Cañizares Fuentes, Rubén Aroca, Miquel Blasco Carlos

**Affiliations:** Instituto de Investigación e Innovación de Salud Integral, Universidad Católica de Santiago de Guayaquil, Guayaquil, Ecuador; Carrera de Medicina, Facultad de Ciencias Médicas, Universidad Católica de Santiago de Guayaquil, Guayaquil, Ecuador; Facultad de Filosofía, Universidad Católica de Santiago de Guayaquil, Guayaquil, Ecuador; Escuela de Nutrición y Dietética, Universidad Espíritu Santo-Ecuador, Guayaquil, Ecuador

**Keywords:** COVID-19, detection, diagnostic tests, symptomatic

## Abstract

**Objectives:**

Evaluate the relevance of the coronavirus disease 2019 (COVID-19) positive case detection policy or model implemented by the Ministry of Public Health (MPH) of Ecuador and to compare it with the experiences of other countries.

**Methods:**

Data contained the daily reports publicized by the MPH. The formulations were carried out under the Conditioned Probability modality applying Bayes’ Theorem. All the COVID-19 tests applied in relation to the confirmed cases per million inhabitants were considered to obtain their level of positivity, and compared with the experience of Iceland and South Korea.

**Results:**

The probability of detecting positive cases of COVID-19 in Ecuador was higher than Iceland and South Korea, because the diagnostic tests were aimed at symptomatic patients, without identifying asymptomatic or mild symptomatic, who play an important role in the transmission of the disease. In addition, many symptomatic patients were examined but will remain undiagnosed due to the unavailability of tests and the low quality of many of them.

**Conclusions:**

The daily reports on the behavior of the COVID-19 issued by the Ecuadorian government do not adequately represent the growth in the number of those infected each day, nor the actual behavior of the epidemic, affecting possible control measures.

In late December 2020, the Chinese government reported the presence of an ongoing outbreak of pneumonia associated with a novel coronavirus, called severe acute respiratory syndrome coronavirus 2 (SARS-CoV-2), in the Wuhan region. On February, the World Health Organization (WHO) named the disease as coronavirus disease 2019 (COVID-19). By the end of March, most countries already had cases of this disease. As part of the control actions, the countries began to carry out diagnostic tests, under the WHO indications. The Ecuadorian Ministry of Public Health (MPH) implemented the application of diagnostic tests, aimed at cases with clinical symptoms compatible with COVID-19.

To face the COVID-19 pandemic, several governments took into account the model described as the “Oxford Study,” which conceives a way of acting to “flatten the curve” (referring to the contagion curve),^1^ which consists of imposing more extreme social withdrawal measures if the income in intensive care units (ICUs) increases, and softening them if they decrease, assuming that sooner or later the majority of the population will develop the infection.

However, it is known of the existence of other models that, in addition to the implementation of social isolation measures, involve the search for the symptomatic population, massively practicing tests to detect the infection in the initial stages.^2^

According to the WHO (2020), any case confirmed by the laboratory, regardless of clinical signs or symptoms, is considered a positive COVID-19 case.^3^ In Ecuador, the case definition is practically the same, but health workers are restricted to applying laboratory tests only to those patients who present symptoms. This measure suggests the systematic exclusion of the asymptomatic population, without it being integrated, in any way, as part of the epidemiological strategy to stop transmission in the community.

On the basis of the above, this article aims to evaluate the relevance of this model of detection of positive cases of COVID-19 implemented by the Ecuadorian MPH and to compare it with those of the other countries that decided to carry out diagnostic tests on asymptomatic patients and suspicious contacts. For this purpose, the information produced by the Ministry of Public Health of Ecuador was used.^4^

## METHODS

The statistical evaluations presented in this article are based on probability analyses, determined using Bayes’ Theorem, where the results are represented with numbers between 0 and 1. The formulations were carried out under the Conditioned Probability modality, that is, probability of an event A occurring when an event B occurs. The estimates were oriented to try to specify the type of relationship between the variables “COVID-19 tests applied” and the “possibility of occurrence of a positive COVID-19 case.” According to WHO,^5^ a case is considered as positive COVID-19 if it is confirmed by laboratory, independent of clinical signs or symptoms.

Bayes’ Theorem is formulated as follows^6^:
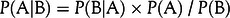
where P(A) is the probability of event A, P(B) is the probability of event B, P (A|B) is the probability of observing event A if B is true; and, P(B|A) is the probability of observing event B if A is true.

In this case, Pa represents COVID-19 confirmed patients with obvious signs of infection, while Pb represents confirmed patients with COVID-19 who had no obvious signs of infection or any type of symptoms.

This gives rise to 2 differentiated approaches that would consist of the observation of:

1. Pa (symptomatic | examined) + Pb (asymptomatic | examined)

2. Pa (symptomatic | examined)

In the first case, it would be based on a principle of randomness (symptomatic + nonsymptomatic), and in the second, selectivity (symptomatic).

With this in mind, the possibility of occurrence obtained in Ecuador, based on the second principle (selectivity), was compared—thanks to the reports issued periodically by the Ecuadorian Ministry of Public Health on COVID-19 positive cases—with the cases of South Korea and Iceland, based on the first principle (randomness), until April 13, 2020.^7,8^

All the COVID-19 tests applied in relation to the confirmed cases per million inhabitants were determined in the 3 countries.

## RESULTS

**FIGURE 1:**
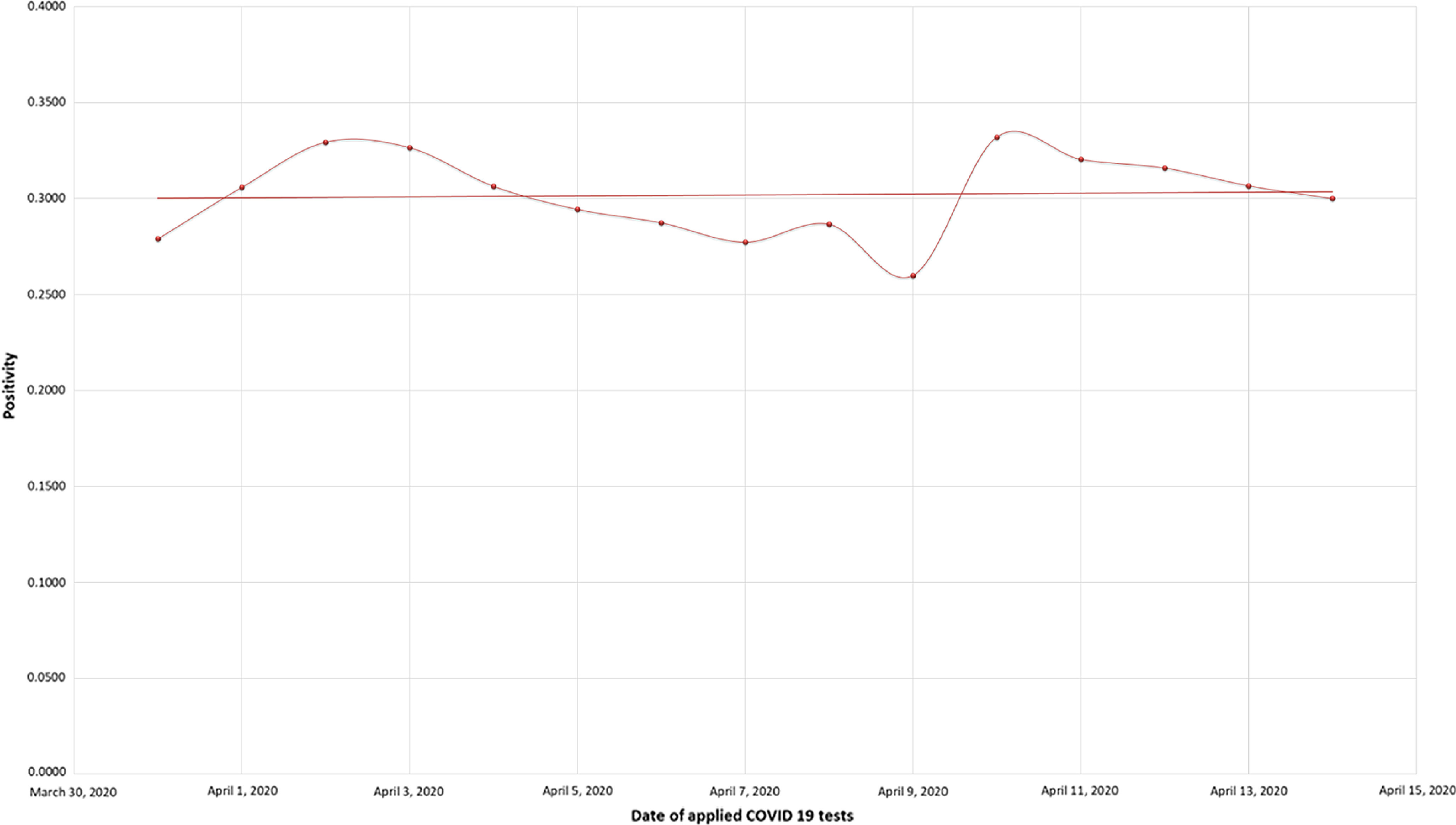
Dates of Applied COVID-19 Tests.

In Ecuador, from March 31 to April 13, 2020, a total of 24,553 COVID-19 tests were carried out, obtaining a total of 7529 COVID-19 confirmed patients.^4^ Taking into account the size of the country’s population (17,468,736 people), it is observed that, between March 31 and April 13, 2020, there was a growth that exceeded 472 test/million inhabitants to 1406 test/million inhabitants (Figure 1). The average positivity found is: Pa = 0.26 ± 0.33.

**FIGURE 2:**
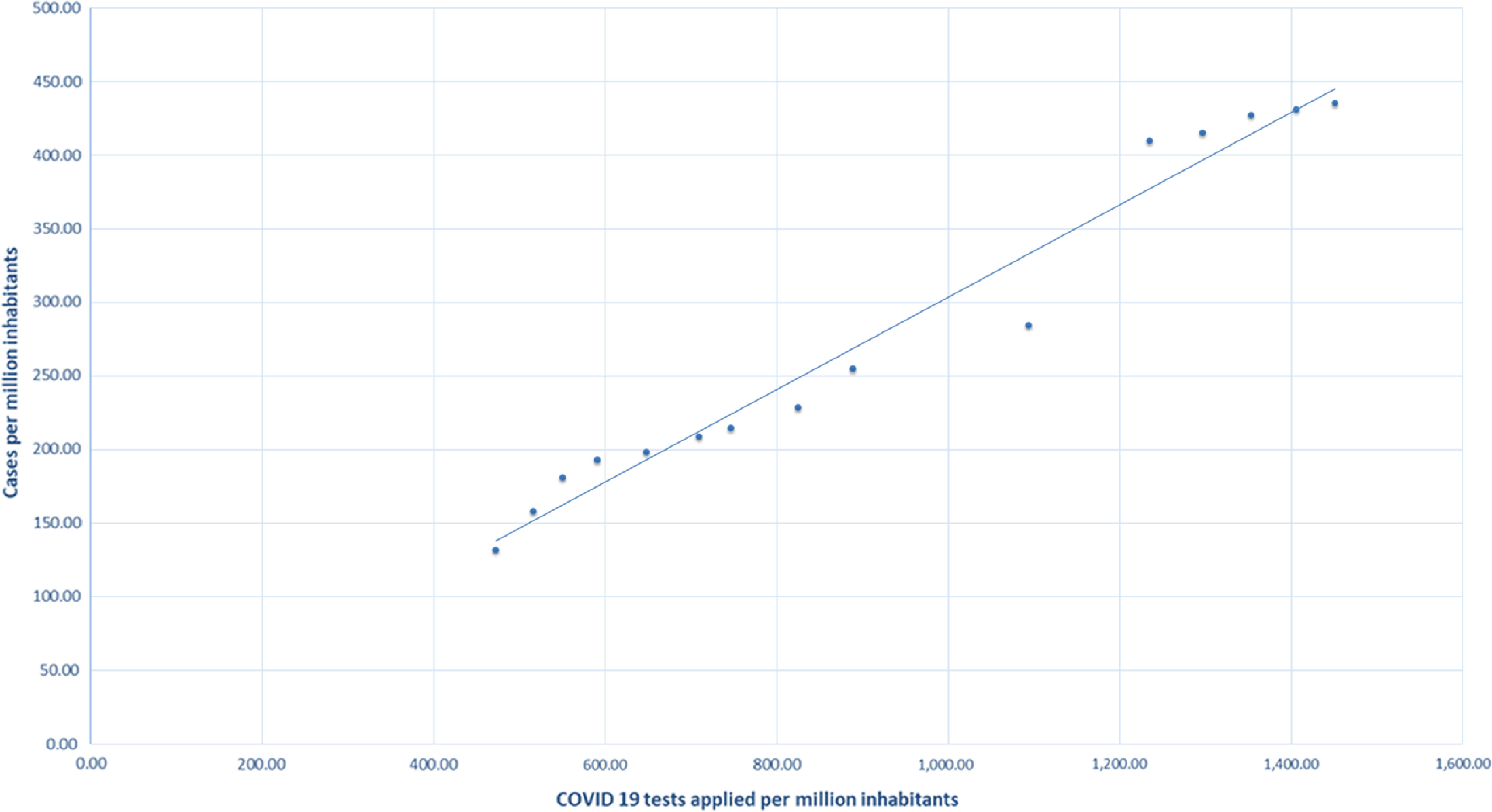
COVID-19 Tests Applied Per Million Inhabitants.

For comparative purposes, this selective approach based on the study of the proportion of confirmed cases of COVID-19 only among the symptomatic (Pa), was compared with the randomized approach used by Iceland and South Korea, which tested both symptomatic and nonsymptomatic (Pa + Pb). As results, during this period, Korea applied 534,552 tests and obtained 10,519 positive COVID-19 cases (Pa + Pb = 0.020); Iceland performed 36,339 tests and obtained 1720 positive cases (Pa + Pb = 0.047); and Ecuador carried out 24,553 tests and obtained 7529 positive cases (Pa = 0.307). When comparing the results of Ecuador with respect to with Korea and Iceland, it is inferred that Pa (symptomatic | examined) has a greater probability than Pb (nonsymptomatic | examined). Thus, in cases with obvious symptoms of COVID-19, a significant correlation with the positivity of the test will be observed more frequently (Figure 2).

## CONCLUSIONS

As seen, the number of positive cases of COVID-19 in Iceland and South Korea are, in relative terms, lower than those observed in Ecuador: 0.05, 0.02, and 0.30, respectively. Although it is evident that the performance of diagnostic tests directed only at symptomatic patients may have a high probability of positivity, this does not contribute to the control of the epidemic at the community level.

In addition, the Ecuadorian public health network had insufficient COVID-19 tests, a condition that is measured as sufficient tests applied for each hundred thousand or million inhabitants and that allows determining the effectiveness and efficiency of the actions of public entities. This meant that the Pa (symptomatic | examined) ratio was not more likely to be met, because many symptomatic patients were examined but were left without a diagnosis. In addition, as seen, Ecuador experienced a supply shortage of synthetic fiber swabs that caused diagnosis disruption.^9^

Lack of testing was not the only issue with SARS-CoV2 surveillance failure, but quality of testing. Regulations for endorsed diagnostic kits in Ecuador are weak, and affect the quality of diagnosis in terms of sensitivity. So far, the positive rate may be even higher as low sensitivity SARS-CoV2 are widely used in this country.^10^

Consequently, the daily reports on the behavior of the COVID-19 issued by the Ecuadorian government do not adequately represent the growth in the number of infected persons each day, nor the actual behavior of the epidemic, affecting possible control measures.
